# Abdominoplasty for male truncal obesity: case report

**DOI:** 10.11604/pamj.2020.36.52.19905

**Published:** 2020-06-01

**Authors:** Ugochukwu Uzodimma Nnadozie, Gabriel Maduwuike Okorie, Charles Chidiebele Maduba, Njoku Isaac Omoke, Amaechi Ugbala, Emmanuel Uchendu, Collins Nwachi Ugwu

**Affiliations:** 1 Department of Surgery, Alex Ekwueme Federal University Teaching Hospital, Abakaliki, Ebonyi State, Nigeria; 2 Department of Surgery, Ebonyi State University/ Alex Ekwueme Federal University Teaching Hospital, Abakaliki, Ebonyi State, Nigeria; 3 Alex Ekwueme Federal University Teaching Hospital/ College of Health Science, Ebonyi state University, Abakaliki, Ebonyi State, Nigeria; 4 Department of Internal Medicine, Alex Ekwueme Federal University teaching Hospital, Abakaliki, Ebonyi State, Nigeria

**Keywords:** Abdominoplasty, elderly male, truncal obesity, quality of life

## Abstract

Truncal obesity and its associated health risk is an enormous burden. The traditional surgical treatment modality is liposuction or lipoabdominoplasty. An uncommon mode of the treatment is the use of abdominoplasty alone or as a surgical component. The aim of this report is to show a satisfactory outcome of abdominoplasty as the only surgical component in the management of severe truncal obesity in elderly male patient. We report a 75 year old Nigerian trader who had truncal obesity with gross abdominal asymmetry and cardiovascular and diabetes mellitus co-morbidities as well as bilateral knee osteoarthritis and social isolation due to truncal disfigurement. He was offered abdominoplasty as a sole surgical option for correction of anterior abdominal wall asymmetry. Apart from post operative wound complications and blood transfusion reactions, the patient had a good recovery and improved quality of life. Abdominoplasty is a rewarding treatment when used as a sole surgical option in centrally obese patients with anterior abdominal wall asymmetry and significant subcutaneous fat thickness.

## Introduction

Truncal obesity or central obesity refers to the abnormal fat distribution in the trunk relative to the rest of the body in an obese patient with BMI ≥ 30 kg/m^2^ as well as in those with normal body mass index [1]. It is an important health concern most commonly found among the middle aged and elderly men [2] and is characterized by excessive subcutaneous and visceral fat mass that more often than not produces an embarrassing beer belly or pot belly effect. The excessive abdominal protrusion with its deforming looks and the psychological distress associated with truncal obesity is an enormous burden. Published reports indicate that truncal obesity correlates strongly with cardiovascular diseases, diabetes mellitus type 2 and dyslipidemias [3,4]. Thus, in addition to this burden, excessive truncal adiposity is a depot that conveys serious health risks. Therefore, treatment of truncal obesity is of medical and aesthetic importance. The mainstay of abdominal contour surgery in men is liposuction/lipoabdominoplasty [5]. In developing countries, aesthetic surgery is under developed [6] and there is paucity of centres with capacity for treatment modality that involves liposuction. Consequently, abdominoplasty is a viable option of abdominal contour surgery in such settings [7,8]. However, published reports indicate preponderance of 30-50 years age group, overwhelming female gender bias and rarity of septuagenarian among patients for abdominoplasty [7-10]. Thus, that a male septuagenarian with a protracted burden of severe truncal obesity sought for aesthetic surgical intervention and had abdominoplasty with a satisfactory outcome and improved quality of life is uncommon in our setting, hence this case report.

## Patient and observation

A 75 year old man who presented with a 15 year history of asymmetrical abdominal enlargement with the right half more greatly enlarged. It started insidiously as a part of generalized weight gain before persisting as a truncal weight excess. There was no symptom of intra-abdominal pathology. He experienced a lot of difficulty with lying prone or supine. He subsequently could not drive his car or walk without a walking stick due to bilateral knee pains and feeling of dragging weight tilt on the right side of the trunk. There was occasional waist pain. He had a positive history of alcohol consumption (an average of 4 bottles of beer per day for 40 years) but stopped drinking 6 years after onset of his symptoms. He also stopped attending social gatherings. He had associated lipoma on the right thigh. He is a known diabetic with hypertensive heart disease on medications. His concerns were both cosmetic and functional. Although the burden was enormous, he was unaware of the availability of aesthetic surgery in our setting till he opened up to a radiologist (during a routine investigation) who refereed him to the plastic surgery clinic. Examination showed an obese looking elderly man in no obvious distress but with a moody affect. His weight was 123 kg and the height was 1.8 metres with a BMI of 38 kg/m^2^. His waist circumference was 145 cm compared to the normal for males which is 102 cm.

Abdominal girth was 141 cm at the umbilicus and 151 cm at the point of maximal enlargement which is 20 cm below the umbilicus. Musculo-skeletal examination showed bilateral knee tenderness without swelling or crepitus. He also had bilateral gynecomastia and lipoma of the right proximal thigh. A diagnosis of truncal obesity in an elderly man with bilateral gynecomastia, early bilateral knee osteoarthritis, hypertension and diabetes was made and patient was worked up for surgery. The ultrasound of the abdominal wall showed variable thickness with the upper quadrants measuring between 7 and 10 cm while the lower quadrants showed a thickness of about 14 cm on the left and 20 cm on the right. The intra-abdominal fat was also sonologically significant. No organ pathology was demonstrated. Laboratory investigations including serum electrolytes, urea and creatinine, fasting blood sugar, complete blood count, serum protein, serum lipids, liver function test and echocardiography, were done and were all within normal ranges. Patient was reviewed by both the cardiologist and the anesthesiologist and certified fit independently. He was counseled and informed consent obtained for abdominoplasty plus or minus liposuction.

Liposuction was later excluded to shorten anaesthesia time and reduce the risk of complications because of his age and co-morbidities. General anesthesia with endotracheal intubation was used after epidural anesthesia failed. About 1.7 litres of Hunstad fluid was also infiltrated essentially for hemostasis and hydro-dissection. Traditional incision for conventional abdominoplasty was used and the protuberant infra-umblical tissue was excised weighing about 8.4 kg. The rectus sheath was plicated in two rows, the umbilicus transposed and the wound closed over two redivac drains, the left in the lower flank and the right in the upper flank. The estimated blood loss was 750 ml and he was transfused two pints of sedimented cells intra-operatively. Post-operative complications included blood transfusion reaction, surgical site infection with partial wound dehiscence. There was periumblical sinus discharging lipoid exudates. All the observed post operative events resolved on wound dressings with povidone iodine and antibiotics.

**Figure 1:**
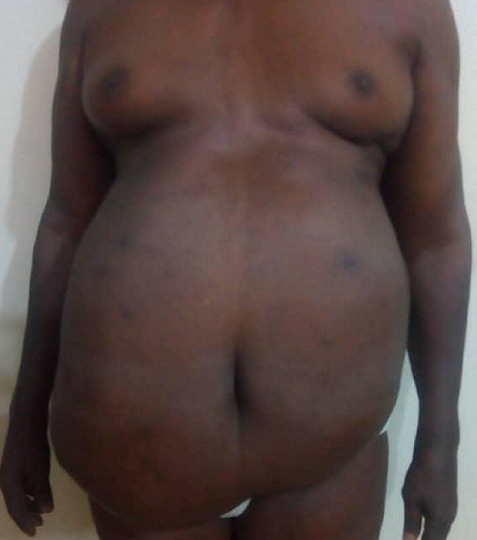
Antero posterior view (pre operative)

**Figure 2:**
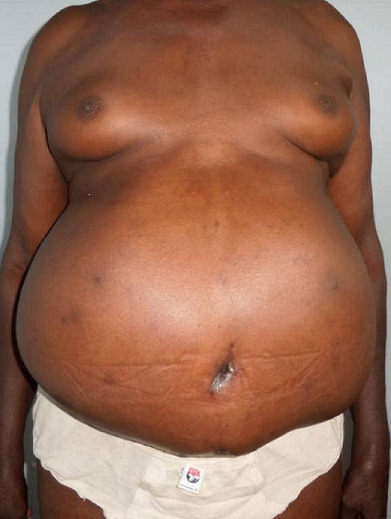
Antero posterior view (post operative)

**Figure 3:**
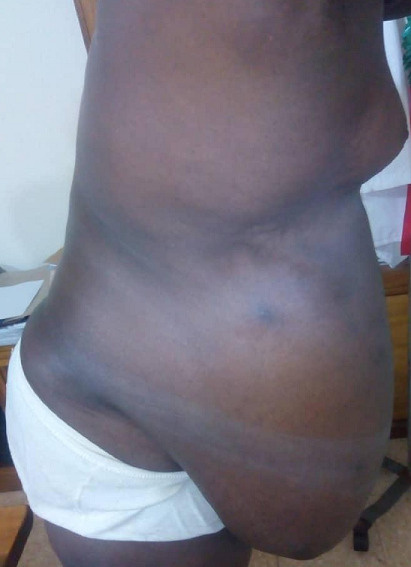
Divers view (pre operative)

**Figure 4:**
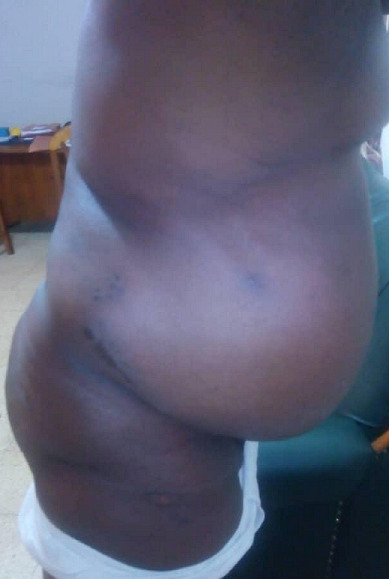
Divers view (post operative)

**Figure 5:**
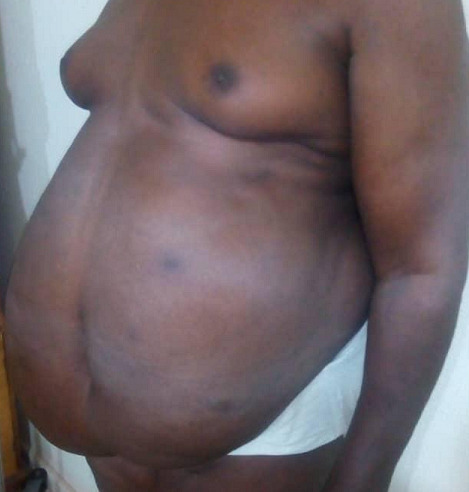
Left anterior oblique view (pre operative)

**Figure 6:**
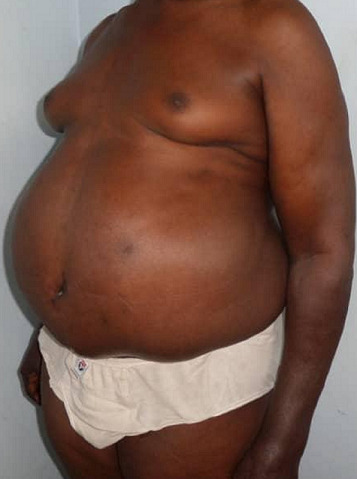
Left anterior oblique view (post operative)

The patient had an amazing improvement in the quality of life and eventual return to his business and social life style. Figure 1, Figure 2, Figure 3, Figure 4, Figure 5 and Figure 6 are different views of images of the patient that shows his pre and post operative looks. He currently walks without a walking stick, drives his car and could use plain trousers and dress corporate, which were previously impossible. He was advised to continue with alcoholic abstinence and dietary control. A year post abdominoplasty, he weighed 116 kg with a BMI of 35.8 kg/m^2^, his waist girth and the abdominal girth at the umblical level were 112 cm and 123 cm respectively. The point of maximal enlargement with a circumference of 125 cm located 10 cm below the umbilicus post-operatively compared to a circumference of 151 cm located at 20 cm below the umbilicus pre-operatively. The post operative ultrasound of the abdominal wall showed variable thickness in a range of 1.3 - 4.4 cm.

## Discussion

In our setting, the level of awareness of availability of aesthetic surgery among the general populace and even among doctors is low [6]. In this case, lack of awareness was the main reason the patient presented for treatment at an age far above the usual age of patients for surgical intervention in truncal obesity. Winoccour *et al.* demonstrated that male sex, BMI > 30Kg/m^2^ and age ≥ 50 years are significant risk factors for complications in abdominoplasty and that the risk of complication in combined procedures is higher than in abdominoplasty alone [10]. All these risk factors for complication were present in this case. Thus, abdominoplasty alone was finally considered the best surgical option of treatment though his consent for abdominoplasty plus or minus liposuction was obtained. The use of abdominoplasty as a sole approach for treatment of central obesity is uncommon. It is more common to employ either liposuction only or lipoabdominoplasty [5]. Abdominoplasty is a procedure of choice for reconstituting the rectus sheath following its diverification especially in multiparous women. In truncal obesity however the abdominal protrusion may be caused mainly by the excessive visceral fat rather than the excessive subcutaneous fat.

But in selected cases where the subcutaneous fat excess is significant cause of abdominal protrusion and medical co-morbidities, abdominoplasty may be a veritable option as has been shown in this case. The presumed cause of truncal obesity is imbalance in diets and energy expenditure in those who are genetically predisposed. Greater consumption of meat is specifically associated with truncal obesity [11]. Alcohol consumption also directly correlates with increased waist circumference and truncal obesity [11]. Thus, to sustain this satisfactory outcome on a long term basis patient was advised to continue with alcoholic abstinence and maintain a balance in his diet and energy expenditure. Treatment approaches have included dietary modifications, exercises, drugs and operative treatment. In men however the visceral fat is more predominant being found twice as much as in the premenopausal women. This limits the place of surgery in treatment of truncal obesity in males. Truncal obesity is defined as absolute waist circumference > 102 cm in men and > 88 cm in women. It is also defined in terms of waist-hip ratio with 0.9 and 0.85 being the respective cut-off for the adult male and female respectively [12,13]. In this case, the outcome of abdominoplasty was very satisfactory to the patient although his post operative waist circumference was 10 cm above normal for men.

## Conclusion

In elderly male patient with truncal obesity, the outcome of abdominoplasty as a sole surgical treatment modality is satisfactory with improvement in overall quality of life.

## Competing interests

The authors declare no competing interests.
